# The impact of delirium on the prediction of in-hospital mortality in intensive care patients

**DOI:** 10.1186/cc9214

**Published:** 2010-08-03

**Authors:** Mark van den Boogaard, Sanne AE Peters, Johannes G van der Hoeven, Pieter C Dagnelie, Pieter Leffers, Peter Pickkers, Lisette Schoonhoven

**Affiliations:** 1 Department of Intensive Care, Radboud University Nijmegen Medical Centre, Nijmegen, P.O. box 9101, Nijmegen, 6500HB, the Netherlands; 2 Department of Epidemiology; NUTRIM/CAPHRI, Maastricht University, P.O. box 5800 Maastricht, 6202AZ, the Netherlands; 3 Scientific Institute for Quality of Healthcare, Radboud University Nijmegen Medical Centre, Nijmegen, P.O. box 9101, Nijmegen, 6500HB, the Netherlands

## Abstract

**Introduction:**

Predictive models, such as acute physiology and chronic health evaluation II (APACHE-II), are widely used in intensive care units (ICUs) to estimate mortality. Although the presence of delirium is associated with a higher mortality in ICU patients, delirium is not part of the APACHE-II model. The aim of the current study was to evaluate whether delirium, present within 24 hours after ICU admission, improves the predictive value of the APACHE-II score.

**Methods:**

In a prospective cohort study 2116 adult patients admitted between February 2008 and February 2009 were screened for delirium with the confusion assessment method-ICU (CAM-ICU). Exclusion criteria were sustained coma and unable to understand Dutch. Logistic regression analysis was used to estimate the predicted probabilities in the model with and without delirium. Calibration plots and the Hosmer-Lemeshow test (HL-test) were used to assess calibration. The discriminatory power of the models was analyzed by the area under the receiver operating characteristics curve (AUC) and AUCs were compared using the Z-test.

**Results:**

1740 patients met the inclusion criteria, of which 332 (19%) were delirious at the time of ICU admission or within 24 hours after admission. Delirium was associated with in-hospital mortality in unadjusted models, odds ratio (OR): 3.22 (95% confidence interval [CI]: 2.23 - 4.66). The OR between the APACHE-II and in-hospital mortality was 1.15 (95% CI 1.12 - 1.19) per point. The predictive accuracy of the APACHE-II did not improve after adding delirium, both in the total group as well as in the subgroup without cardiac surgery patients. The AUC of the APACHE model without delirium was 0.77 (0.73 - 0.81) and 0.78 (0.74 - 0.82) when delirium was added to the model. The *z*-value was 0.92 indicating no improvement in discriminative power, and the HL-test and calibration plots indicated no improvement in calibration.

**Conclusions:**

Although delirium is a significant predictor of mortality in ICU patients, adding delirium as an additional variable to the APACHE-II model does not result in an improvement in its predictive estimates.

## Introduction

Predictive models are widely used in ICUs to estimate the disease severity and estimate the risk of death or to identify patients at high risk of dying [1]. Predictive estimates are important from both a clinical and administrative perspective. These estimates can be used to inform patients and their families about likely outcomes [1,2], to monitor response to treatment, to guide physicians in making clinical decisions [2], and to monitor or compare the performance of different ICUs [3]. A commonly used prediction model in the ICU is the Acute Physiology and Chronic Health Evaluation (APACHE)-II [4], which is measured within 24 hours of ICU admission. Importantly, although the APACHE-II score was developed in the early 1980s, it still represents the most widely used predictive model to estimate in-hospital mortality and it remains a valid measure of severity of illness. The APACHE-II is able to correctly differentiate between patients who are and who are not at risk of dying in 62% to 88% of patients [5]. The Glasgow Coma Scale is the only variable referring to brain (dys)function in the APACHE-II score [4]. Delirium, another brain disorder, is not included in the APACHE-II model, despite its high incidence rate in ICU patients and the growing evidence of its association with poor outcomes such as increased morbidity and mortality rates, and prolonged length of hospital stay [6,7].

Delirium, defined as a disturbance of consciousness and cognition that develops over a short period of time and fluctuates over time, is induced by an underlying physical cause such as the development of severe medical illness, co-morbidities and changes in drug use [8,9]. One-third of patients are delirious on initial assessment, and the majority who develop delirium do so within 48 hours of admission [8].

Consequently, adding delirium to the existing APACHE-II model could improve the predictive estimates. However, despite the strong association between delirium and mortality, such an association does not necessarily imply clinical relevance or better prediction.

The aim of our study was to evaluate whether delirium, if present within 24 hours after ICU admission, improves the predictive accuracy of the APACHE-II score of in-hospital mortality of critically ill patients.

## Materials and methods

This prospective cohort study was carried out in the Radboud University Nijmegen Medical Centre, the Netherlands. This is a 960 bed university hospital with 33 ICU beds for adults where annually 2,000 to 2,500 (cardiothoracic surgery, neurosurgical, medical, surgical and trauma) ICU patients are admitted. The study was approved by the local Medical Ethical Committee, which waived the need for informed consent because no interventions were carried out.

Consecutive adult patients admitted to the ICU between February 2008 and February 2009 were included. Patients were excluded when they had a sustained Richmond agitation sedation score (RASS) of -4/-5, length of stay on the ICU for 12 hours or less, had serious auditory or visual disorders, were unable to understand Dutch, were severely mentally disabled or suffered from receptive aphasia.

To detect delirium, all patients were screened with the validated Dutch version of the Confusion Assessment Method-ICU (CAM-ICU) [10]. The assessment with the CAM-ICU was performed three times per day by well trained ICU nurses during the patient's entire ICU stay [11]. For this study patients were diagnosed with delirium when they had a minimum of one positive CAM-ICU screening assessment. As for the other parameters used in the APACHE-II score, we used delirium that occurred within 24 hours after ICU admission. Demographic, laboratory, clinical data, and hospital mortality were collected. Naturally, various risk factors for the development of delirium may differ between patients, but these were not registered because the aim of the present study was merely to investigate if the predictive value of the APACHE-II score improved when delirium, irrespective of its cause, was added.

As the APACHE-II was originally not validated for cardiac surgery patients, a subgroup analysis was also performed without cardiac surgery patients.

### Statistical analysis

Patient characteristics at baseline and the incidence of delirium within 24 hours, and in-hospital mortality were evaluated. Normally distributed data were tested parametrically using the Student's T-test, and not normally distributed data were tested non-parametrically using the Mann-Whitney U test. The correlation between delirium and the APACHE-II score was tested using the Spearman's rho. The association between delirium and in-hospital mortality was evaluated in a univariate and multivariate logistic regression model. The first model consisted of patient's overall score on all variables of the APACHE-II score as the only predictive variable. The second model, based on the data of the same patients, consisted the variables of the APACHE-II score with delirium added as a new predictor. Differences in model performance between the APACHE-II model with and without delirium were estimated on discrimination (area under the receiver-operating-characteristic (AUC) curve). The two AUCs were compared using the z-statistic for comparing AUCs derived from the same cases as described by Hanley and McNeil [12]. A z-value between -1.96 and +1.96 was considered as there being no significant differences between the two AUCs and with the most common used features on calibration (Hosmer-Lemeshow's goodness-of-fit and calibration plots). A two-sided significance level of 5% and 95% confidence intervals (CI) were used for statistical inference. Statistical analysis was performed using SPSS 16.01 and MedCalc^® ^version 11.3.1.0 (MedCalc Software, Mariakerke, Belgium).

## Results

During the study period, 2,116 patients were admitted to the ICU of whom 376 patients were excluded, leaving 1,740 patients for outcome analysis. The main reason for exclusion was persistent coma (36%) that made the detection of delirium impossible. Baseline characteristics of the included patients, with and without delirium within 24 hours, are shown in Table 1, and baseline characteristics of the patients, with and without cardiac surgery patients, are shown in Table 2. A total of 332 patients (19%) were delirious, 132 at the time of admission and 200 within 24 hours after admission. The overall in-hospital mortality rate was 7.7%. In the non-delirious group 80 of 1,408 patients (5.7%) died, and in the delirious group this was 54 of 332 patients (16.2%).

**Table 1:** Baseline characteristics and differences of delirious (within 24 hours after ICU admission) and non-delirious patients*

	Non-delirium < 24 hours	Delirium < 24 hours	*P*-value
Age in years	61 ± 15	66 ± 14	0.11
Male, N (%)	134 (21.1)	198 (17.9)	0.08
APACHE-II score	14 ± 6	17 ± 6	0.18
Length of stay-ICU in days (median-IQR)	1 (1-3)	3 (1-9)	< 0.0001
Length of stay-hospital in days (median-IQR)	8 (5-16)	15 (8-33)	< 0.0001
Urgent admission, N (%)	708 (50.3)	253 (76.2)	< 0.0001
ICU admission type (%):			
- Surgical	910 (87.7)	127 (12.2)	
- Medical	302 (70.1)	129 (29.9)	
- Trauma	66 (78.6)	18 (21.4)	
- Neurology/neurosurgical	130 (69.1)	58 (30.9)	
Died, N (%)	80 (5.7)	54 (16.2)	< 0.0001

APACHE, Acute Physiology and Chronic Health Evaluation-II; IQR, interquartile range.

* Data are presented as mean ± standard deviation, unless otherwise mentioned.

**Table 2:** Baseline characteristics of the patients in the total group and the subgroup without cardiac surgery patients*

	Total group *n *= 1740	Non-cardiac surgery patients *n *= 881
Age in years	62 ± 14	58 ± 16
Gender M/F, N	1109/631	506/375
APACHE-II score	15 ± 5	16 ± 6.6
Length of stay-ICU in days (median-IQR)		
- non delirious (within 24 hours after admission)	1 (1-3)	2 (1-7)
- delirious	3 (1-9)	3 (2-10)
Length of stay-hospital in days (median-IQR)		
- non delirious (within 24 hours after admission)	8 (5-16)	14 (8-27)
- delirious	15 (8-33)	19 (10-36)
Urgent admission, N (%)	961 (55.2)	703 (82.8)
ICU admission type (%):		
- Surgical	59.6	23.4
- Medical	24.8	45.9
- Trauma	4.8	9.4
- Neurology/neurosurgical	10.8	21.3
Delirium, N (%)	332/1740 (19.1)	223/881 (25.3)
- before admission	132 (7.6)	106 (12.0)
- within 24 hours after admission	200 (11.5)	117 (13.2)
Died, N (%)		
- non delirious (within 24 hours after admission)	80 (5.7)	71 (10.8)
- delirious	54 (16.2)	36 (16.1)

APACHE, Acute Physiology and Chronic Health Evaluation-II; F, female; IQR, interquartile range; M, male.

* Data are presented as mean ± standard deviation, unless otherwise mentioned.

**Figure 1:**
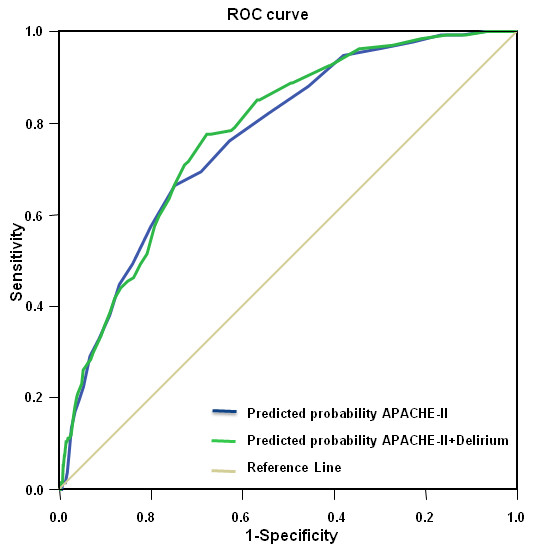
**Receiver-operating-characteristic and the area under the curve of different prediction models with and without delirium**. APACHE-II, Acute Physiology and Chronic Health Evaluation-II.

**Figure 2:**
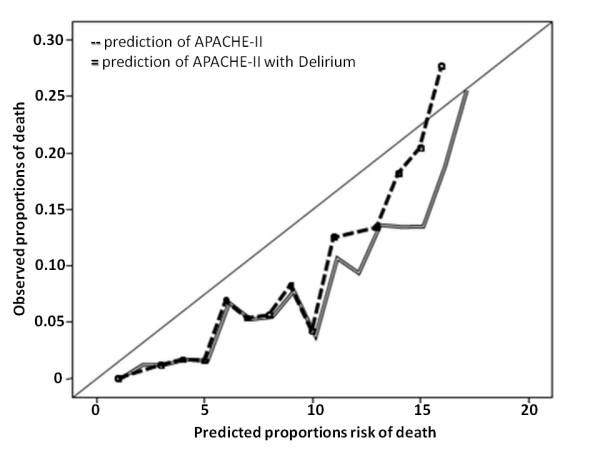
**Calibration plots of the APACHE-II model and of the APACHE-II model with delirium**. APACHE-II, Acute Physiology and Chronic Health Evaluation-II.

The crude odds ratio (OR) of the presence of delirium within 24 hours of ICU admission and in-hospital mortality was 3.22 (95% CI: 2.23 to 4.66), and between the APACHE-II score and in-hospital mortality was 1.15 (95% CI: 1.12 to 1.19) per APACHE-point. The AUC of the APACHE-II model without delirium was 0.77 (95% CI: 0.73 to 0.81, standard error 0.19) and 0.78 (95% CI: 0.74 to 0.82, standard error 0.19) when delirium was added. Comparison of the two AUCs with the Hanley and McNeil test resulted in a z-value of 0.92 (*P *= 0.36) indicating that both AUCs not significant different and that addition of delirium to the APACHE-II score does not result in an improvement in discriminative power (Figure 1). Calibration plots (Figure 2) and the Hosmer-Lemeshow test (HL-test) showed a decrease of calibration after adding delirium to the APACHE-II score (HL-test chi-square 12.38 and after adding delirium to the APACHE-II chi-square 17.93). The Spearman's rho correlation between delirium and the APACHE-II score was 0.22 (*P *< 0.0001). The subgroup, in which cardiac surgery patients were excluded, consisted of 881 patients. The crude OR for delirium present within 24 hours after ICU admission and in-hospital mortality in this subgroup was 1.59 (95% CI: 1.03 to 2.46) and for APACHE-II and in-hospital mortality was 1.11 (95% CI: 1.08 to 1.15) per point.

## Discussion

The main finding of the present study is that, although delirium present within 24 hours after ICU admission, is associated with increased in-hospital mortality, adding delirium to the APACHE-II score does not improve its accuracy in predicting in-hospital mortality. Similar results were obtained in a subgroup analysis of non-cardiac surgery patients.

The availability of an easy to use instrument that needs a limited amount of variables to predict the outcome of ICU patients is of great importance for clinical ICU practice. The APACHE-II score represents such an instrument with a moderate predictive value for in-hospital mortality. Comparable with previous reports [5] we found an AUC of the APACHE-II of 0.77. Theoretically, adding a prevalent and relevant variable to the APACHE-II score could improve its predictive value. Delirium could represent such a variable, because it is a frequent and serious disorder on the ICU associated with poor patient outcome. Although our study confirms previous reports [6], showing that the presence of delirium is an independent risk factor for mortality, we demonstrate that the addition of delirium does not improve the predictive value of the APACHE-II score. There are several reasons why adding a new predictive variable may not result in a better accuracy of a predictive model including a low prevalence of the variable, the absence of predictive value of this variable, and the presence of a correlation between the predictive variable and the variable(s) originally included in the predictive model (i.e. APACHE-II). We showed that prevalence and predictive value of the presence of delirium are adequate. However, although the occurrence of delirium in critically ill patients is an independent risk factor for mortality [6], we found that the APACHE-II score correlated significantly with the occurrence of delirium within 24 hours. As a consequence, delirium has no additive effect in the predictive value of the APACHE-II score.

Importantly, our data do not exclude a possible additive effect of incorporating delirium in models that are not focused on the first 24 hours of ICU stay, such as the sequential organ failure assessment (SOFA) score. Although there is a statistically significant association between delirium present within 24 hours after ICU admission and APACHE-II score, an association of 0.22 is rather low. Probably residual confounding plays an important role. The effect of adding delirium to dynamic predictive models such as the SOFA score, warrants further investigations because in a substantial part of the patients delirium is detected after the first 24 hours after ICU admission as a result of worsening of their clinical situation.

## Conclusions

An independent association was found between delirium present within 24 hours after ICU admission and in-hospital mortality. However, adding delirium as a predictive variable to the APACHE-II score did not improve its predictive value.

## Key messages

• Delirium in ICU patients present within 24 hours after ICU admission, is associated with increased in-hospital mortality.

• Adding delirium to the APACHE-II score does not improve its accuracy in predicting in-hospital mortality.

## Abbreviations

APACHE-II: Acute Physiology and Chronic Health Evaluation-II; AUC: area under the receiver operating characteristics curve; CAM-ICU: Confusion Assessment Method-Intensive Care Unit; CI: confidence interval; HL-test: Hosmer-Lemeshow test; OR: odds ratio; RASS: Richmond Agitation Sedation Score; SOFA: Sequential Organ Failure Assessment.

## Competing interests

The authors declare that they have no competing interests.

## Authors' contributions

MvdB and SP both carried out the study, the statistics and drafted the manuscript. PD and PL participated in the statistical analysis and helped to draft the manuscript. JvdH and PP supervised the study and helped to draft the manuscript. LS participated in the study design, supervision and to draft the manuscript. All authors read and approved the final manuscript.
